# Living With Bipolar Disorder in the Time of Covid-19: Biorhythms During the Severe Lockdown in Cagliari, Italy, and the Moderate Lockdown in Tunis, Tunisia

**DOI:** 10.3389/fpsyt.2021.634765

**Published:** 2021-02-24

**Authors:** Mauro Giovanni Carta, Uta Ouali, Alessandra Perra, Azza Ben Cheikh Ahmed, Laura Boe, Amina Aissa, Stefano Lorrai, Giulia Cossu, Alessandro Aresti, Antonio Preti, Fethi Nacef

**Affiliations:** ^1^Department of Medical Sciences and Public Health, University of Cagliari, Cagliari, Italy; ^2^Department of Psychiatry A, Razi Hospital, Tunis, Tunisia; ^3^Faculty of Medicine of Tunis, University of Tunis El Manar, Tunis, Tunisia; ^4^Department of Neuroscience, University of Turin, Turin, Italy

**Keywords:** bipolar disorder, lockdown, biorhythms, COVID-19, depressive episodes

## Abstract

**Background:** Restrictions during Covid-19 pandemic lockdown, in which rhythms of life have been compromised, can influence the course of bipolar disorder (BD). This study follows patients with bipolar disorder living in two geographically close cities (Cagliari and Tunis), but with different lockdown conditions: less severe in Tunis.

**Methods:** Two cohorts were evaluated during lockdown (April 2020, t0) and 2 months later with lockdown lifted for a month (t1). Individuals were: over 18 years old without gender exclusion, BD I or II, in care for at least 1 year, received a clinical interview in the month before the start of the lockdown, stable clinically before the lockdown. The assessment was conducted by telephone by a psychiatrist or psychologist with good knowledge of patients. Diagnoses were made according to DSM-5 criteria. Depressive symptoms were collected through the Hamilton Rating Scale for Depression; cut-off 14 indicative of depressive episode. Circadian rhythms were measured using the BRIAN scale.

**Results:** Forty individuals in Cagliari (70%female, age 48.57 ± 11.64) and 30 in Tunis (53.3% Female, age 41.8 ± 13.22) were recruited. In Cagliari at t0 45% had depressive episodes against none in Tunis, a similar difference appeared at t1. At t0 and t1 the Cagliari sample had more dysfunctional scores in the overall BRIAN scale and in the areas of sleep, activities and social rhythms; no differences were found in nutrition, both samples had predominantly nocturnal rhythm. In Cagliari at t0 and t1, the depressive sub-group showed more dysfunctional scores in the BRIAN areas sleep, activity, and nutrition. However, the differences in biological rhythms resulted, through ANCOVA analysis, independent of the co-presence of depressive symptoms.

**Discussion:** A rigid lockdown could expose people with BD to depressive relapse through dysregulation of biological rhythms. The return to more functional rhythms did not appear 1 month after lockdown. The rekindling of the pandemic and the restoration of new restrictive measures will prevent, at least in the short term, the beneficial effect of a return to normality of the two cohorts.

This was a limited exploratory study; future studies with larger samples and longer observational time are needed to verify the hypothesis.

## Introduction

The Covid-19 outbreak emerged in China at the end of December 2019, some weeks afterwards the epidemic reached other Asian countries and then Europe and the Americas (1); the World Health Organization declared the pandemic on 11 March 2020 (2). A lockdown was declared to contain the rapid spread of this virus in almost all the countries (3). Italy was one of the most affected nations and had to undergo an immediate very hard lockdown (4). Tunisia was not hit hard initially (5).

The pandemic and subsequent lockdown has produced drastic changes in people's lives. Several studies have found a tremendous psychosocial impact on communities with higher prevalence rates of anxiety, depression, stress-related symptoms, increase in alcohol consumption associated with the COVID-19 pandemic in different countries (6–8).

Lockdown and isolation to counter spread of the disease reduced new cases but produced, in addition to fear and anxiety related to the risk of becoming infected and stress on seeing infected relatives and friends, a negative impact on psychological well-being, with feelings of loneliness and anger (9, 10). A recent study carried out in Italy (8) found greater symptoms of depression with circadian rhythm dysregulation.

A study carried out on a sample of people affected by bipolar disorder in Australia “suggests a degree of resilience in BD patients; despite large pandemic related increases in subjective cognitive dysfunction” (11), in contrast, a recent review found individuals with bipolar disorder at risk of poorer physical and mental health outcomes during the outbreak owing to the lack of health and emotional support (12). Authors speculated that “some risk factors associated with bipolar disorder, including irregular social rhythms … may be compounded by lockdowns, social isolation” (12).

Bipolar disorder is a common, recurrent condition which severely impacts the life of people living with it; currently it is considered one of the leading causes of disability in the world (13, 14).

Alterations of the circadian rhythm are a central element of bipolar disorders and have been implicated in the genesis of the illness (7, 15, 16). Concomitantly, there is a strong vulnerability in these disorders to the alteration of the rhythms induced by stimuli, specifically by alteration of light conditions (17). The circadian rhythmicity generated by the biological clock structures the functioning of human beings on a 24-h cycle. This clock is adjusted daily by internal and external signals, of which light is the most influential. It has in fact been hypothesized that the socio-cultural changes that have led to a total modification of light patterns in the modern metropolis may be a reason for the increase in bipolar disorders in our society, and that it may determine an adaptive condition of certain “subthreshold” forms (18).

The hypothesis of our study is that restrictions during lockdown put in place to counter the Covid-19 pandemic, in which the interplay of daily habits and rhythms of life are necessarily compromised, can influence the course of bipolar disorder. To attempt verification of the hypothesis, we decided to consider the state of circadian rhythms and mood conditions in two cohorts of patients with bipolar disorder having comparable courses and living in two geographically close cities (Cagliari and Tunis), but with different lockdown conditions: very strict in the Sardinian, Italian city in contrast to less severe in Tunis. The two cohorts were evaluated during the first epidemic expansion, and in the subsequent phase of quiescence and lifting of the lockdown.

The aim was to measure whether circadian rhythms were more altered in Cagliari, and if mood conditions differed between the two cities in the sense of higher frequency of depressive episodes in Cagliari in association with the more severe lockdown.

## Methods

### Study Design

This is a study on two cohorts of patients with bipolar disorder recruited in Cagliari, Sardinia, Italy and Tunis, Tunisia. The two cohorts were evaluated during the lockdown in the maximum expansion of the first epidemic wave in Sardinia and Tunisia (April 2020) and 2 months later (June 2020) when the lockdown had been lifted for a month both in Cagliari and in Tunis.

### Setting

The two cohorts were recruited, one at the Center for Psychiatry of Consultation and Psychosomatics of the University of Cagliari, the other at the Outpatient Clinic of Department Psychiatry A of Razi Hospital La Manouba situated in the Greater Tunis area. The metropolitan areas of Tunis and Cagliari are relatively close geographically (Cagliari is about 200 km further north and slightly west), enjoy the same mild Mediterranean climate and amounts of sunlight. However, at the time of the first evaluation, Cagliari was subjected to a very strict lockdown (19): people were permitted to leave their homes solely for health purposes or to buy food, all shops except those for essential items were closed, practically all offices were closed and so operated remotely. In Tunis, the full lockdown was imposed for a shorter period, and was less enforced than in Italy. Although people were required to stay home and leave the house only to buy food or for seeking medical help, it was tolerated to move within a town or the district of a city, or to meet people individually in private or public, and many other services remained available for use (20–22).

The impact of the pandemic was substantially different in Italy and Tunisia. On April 30, 2020, in Tunisia there were only 4.67 cases × 100,000 inhabitants and 40 deaths in total (0.3 × 100,000) due to Covid-19, in Italy there were 338.6 cases × 100,000 inhabitants and 27,967 deaths (46.3 × 100,000) (23). However, specifically in Sardinia, frequencies far below the national average and not very discordant from neighboring Tunisia were recorded at the time.

The assessment was conducted by telephone (given the limitations of the lockdown) by health professionals (psychiatrist and psychologist) who had a good knowledge of the patients.

### Sample

The two cohorts consisted of individuals who had the following characteristics: Age over 18 without gender exclusion, Diagnosis of Bipolar Disorder Type I or II, had been in care at one of the centers for at least 1 year, had received a clinical interview in the month before the start of the lockdown, had been found to be clinically stable at the last clinical assessment before the lockdown. Individuals who had presented with a depressive or manic episode at the time of the last checkup were excluded.

Table 1 reports the description of the sample at the first evaluation in the two cohorts.

**Table 1:** Study Sample.

	**Cagliari**	**Tunis**	*Chi-square with df=1, or °F (ANOVA) with df=68	***p***
Age	48.57 ± 11.64	41.8 ± 13.22	°5.161	0.026
Female	28 (70%)	16 (53.3%)	*2.040	0.153
Bipolar II	26 (65%)	17 (56.6%)	°0.502	0.478
Mood Stabilizers (Lithium, Valproate or Lamotrigine)	29 (96.6)	38 (95%)	0.116	0.703
Antipsychotics	21 (70%)	20 (50%)	0.259	0.107
Antidepressants	1 (3.3%)	6 (15%)	0.148 (with Yates correction)	0.227

### Study Tools

Psychiatric diagnoses were conducted according to the DSM-5 criteria by experienced psychiatrists. Depressive symptoms were collected through the Hamilton Rating Scale for Depression (HAM-D) (24); scores > 14 were considered indicative of a depressive episode. We decided to investigate only the occurrence of depressive episodes rather than manic ones at first because the incidence of manic episodes is notoriously less frequent than that of depressive episodes.

In accordance with previous research, we calculated that owing to the short observational period (2 months) and the sample size of the two groups (40 individuals in the group exposed to rigid lockdown, 30 individual not exposed), verification of a difference in the conceivable onset of manic episodes would not have been possible. The average time spent ill by people with bipolar disorder is 44% in BPI, and 43% in BPII; of this time in depression is 70%in BP I, and 81% in BPII. This means that BPIs spend 30.8% of the course of the illness in depression and 13.2% in mania or a mixed state (a large part of previous mixed state is now classified as depressive episode with mixed symptoms). BPIIs spend 34.8% in depression and 8.7% in mania or mixed state (25).

A study conducted in Scandinavia comparing two cohorts of patients with bipolar disorder for 7 years even larger than ours and subdivided into exposure or non-exposure to an important risk factor for relapses (having a personality disorder) failed to find a statistically significant difference (26). By recalculating the power of the study on the basis of the differences found, in this specific case a sample of about 160 controls for 7 years would have been necessary to avoid a beta error. The present study is merely exploratory; consequentially, we have no idea what differences to expect. But intuitively, even assuming that the difference is very large, an immense sample would be needed to highlight a difference in mania. It would certainly be much easier to highlight a possible difference in depressive episodes. Given the particular framework in which the study took place, it was chosen not to stress the participants more than necessary with structured interviews/tools. For clinical and ethical reasons (not for research), a screening for manic episodes was conducted, but in a colloquial way, with questions specifically targeted to the individual and in an unstructured and standardized way. It is known that screening questionnaires (27) and structured interviews (28) are not wholly accurate in identifying manic episodes.

Circadian rhythms were measured using the BRIAN scale (29) in the Italian version already translated and validated (30) and in the adapted Tunisian Arabic version currently being validated. BRIAN was designed to be administered by a qualified health professional; the time of observation refers to the last 14 days before the assessment. The 18 items evaluating sleep (difficulty falling asleep, difficulty waking up, being satisfied with the hours of sleep), activity (difficulty completing the usual activities such as house cleaning, shopping and work, study, or leisure activities), social rhythm (difficulty interacting with people and not overusing TV and the internet), eating patterns (difficulty respecting eating habits, meal times and quantities of food) and dominant rhythm (nocturnal or diurnal). All items were rated using a 4-Likert point scale scoring 1 = not at all, 2 = rarely, 3 = sometimes, 4 = often, or in some items 1 = never, 2 = rarely, 3 = often, 4 = always. The overall score was obtained by adding the scores of each item.

The lower the score indicated, the higher the level of adjustment to biological rhythms. Some items needed to be reversed to calculate the score.

### Data Analysis

Comparison of the frequencies of depressive episodes (i.e., positive to the screening test for depression—Hamilton Depression Rating Scale with 14 as cut-off) between Cagliari and Tunis and between t0 and t1 was conducted through the chi-square test with Yates correction when required. To verify if the frequency of depressive episodes we found were homogeneous with respect to normative data, we compared, calculating the Relative Risk and CI 95%, the incidence of depressive episodes in 2 months in the two cohorts considered with the incidence of depressive episodes in 2 months found in a cohort made up of 1,135 individuals in a recent prospective study aimed at identifying recurrence determinants (31).

One-way repeated-measures analysis of variance (ANOVA) was used to analyze the effects of the change over time of alterations in biological rhythms in each town (average score achieved on the BRIAN scale and in each dimension of the scale). The Mauchly test of sphericity was used to test the assumption of the sphericity of the test statistic. The effect size of the differences was reported using partial η^2^.

Analysis of covariance (ANCOVA) was applied to investigate whether alterations in biological rhythms (score achieved on the BRIAN scale) were merely the results of greater occurrence of depression or there was an independent effect of the impact of lockdown on the lifestyle of patients in Cagliari and in Tunis.

To further explore the role of each dimension of the BRIAN scale, while avoiding multiple comparisons, we used repeated-measures multivariate analysis of variance (MANOVA). Data were analyzed using general linear models (GLM) MANOVA with Wilks' Lambda and Greenhouse-Geisser adjustments. We tested the effects of the location (Cagliari vs. Tunis), the effects of time (baseline and 3-month follow-up), and the group-by-time interaction for both the five dimensions (sleep, activity, sociality, eating, global rhythms) of our global measure of alterations of biological rhythms, the BRIAN scale. The threshold for a statistically significant result was set at *p* < 0.05.

All analyses were carried out with the SPSS version 22.0 software (IBM SPSS Statistics, Chicago, IL).

### Ethical Aspects

During the first phone call, the researchers provided exhaustive information concerning the objectives of the study and informed the patient about the possibility of discontinuing the interview if they wished. The researchers also explained that the data would be collected by an anonymous database for confidentiality. Each participant formulated an explicated informed consent. The study was carried out according to the Helsinki Declaration.

The ethical committee of the Azienda Mista Ospedaliero Universitaria di Cagliari approved the study as well as the ethics committee of Razi Hospital in Tunis.

## Results

Eighteen of the 40 people evaluated in Cagliari at the first evaluation (t0) had a Hamilton score higher than 14, against none of the people evaluated in Tunis (45 vs. 0%, χ2 = 23.721; *p* < 0.0001), the data were substantially repeated at t1 after the lockdown with 14 Sardinians who had a score of > 14 and no Tunisians (35 vs. 0%, χ2 = 14,318; *p* < 0.0001). The decreasing trend in the frequencies of Depressive Episodes in the Sardinian sample between t0 and t1 did not reach statistical significance (45 vs. 35%, χ2 = 0.833; *p* = 0.361). In the cohort considered as normative (31), after 2 months there was an incidence of 15 depressive episodes in 1,135 patients. In the Tunis cohort we had 0 depressive episodes in 30 patients in the same time, so the Relative Risk (RR) calculated with respect to the Eitan Cohort was = 0 (95% CI from 0 to 11.85), chi square with Yates correction = 0, *P* = 1. In the Cagliari cohort we had an incidence of 18 depressive episodes out of 40 patients in 2 months, the RR was 23.48 (95% CI from 11.8 to 46.26) and the chi-square with the Yates correction was 170.2, *p* < 0.0001.

Table 2 shows the comparison between the Sardinian and Tunisian samples in the mean ± standard deviation scores of the five specific areas and of the total score of the BRIAN scale at t0 and t1. It was observed that during the lockdown the Cagliari sample had more dysfunctional scores in the areas of sleep, activities and social rhythms and no differences were found in nutrition. In the area of dominant rhythm (day/night rhythm), the Cagliari and Tunisian samples both had a predominantly but not markedly nocturnal rhythm which, on the BRIAN scale, is coded with higher scores considered more dysfunctional. The total score of the scale shows a more dysfunctional result in the Cagliari sample.

**Table 2:** BRIAN scale and sub-scale scores during and after the lockdown in the Cagliari and Tunisian samples.

	**Sleep**	**Activity**	**Social**	**Nutrition**	**Rhythms**	**Total**
Cagliar duringi	12.4 ± 3.92	11.3 ± 4.03	7.67 ± 2.77	7.50 ± 2.98	5.32 ± 2.33	44.1 ± 10.83
Cagliar after	12.3 ± 3.55	11.8 ± 4.80	7.72 ± 2.58	7.78 ± 3.55	5.82 ± 2.16	45.07 ± 12.00
Tunis during	7.97 ± 3.11	7.40 ± 2.47	5.47 ± 1.93	7.00 ± 2.44	5.46 ± 2.28	34.8 ± 8.05
Tunis after	8.39 ± 3.17	7.3 ± 2.04	5.30 ± 1.82	7.03 ± 2.20	5.53 ± 2.35	35.4 ± 8.15
*F*_(1, 68)_, df	28.148	24.665	15.417	0.596	0.063	19.925
*P*	<0.0001	<0.0001	<0.0001	0.443	0.803	<0.0001

**Table 2A:** Multivariate results of MANOVA of differences by time in biological rhythms in Cagliari and Tunis.

	**Wilk's Λ**	**Multivariate statistics**	**Partial η^2^**
Town	0.453	*F*_(5, 64)_=15.5; *p* < 0.0001	0.547
Time	0.510	*F*_(5, 64)_=12.3; *p* < 0.0001	0.490
Time × Town	0.527	*F*_(5, 64)_=11.5; *p* < 0.0001	0.473

**Table 2B:** Univariate results of MANOVA of differences by time in biological rhythms in Cagliari and Tunis.

	**Greenhouse-Geisser corrected statistics**	**Partial η^2^**
Sleep		
Town	*F*_(1, 68)_ = 34.9; *p* < 0.0001	0.339
Time	*F*_(1, 68)_ = 0.6; *p =* 0.425	0.009
Time × Town	*F*_(1, 68)_ = 1.1; *p =* 0.301	0.016
Activity		
Town	*F*_(1, 68)_ = 39.7; *p* < 0.0001	0.369
Time	*F*_(1, 68)_ = 0.2; *p =* 0.684	0.002
Time × Town	*F*_(1, 68)_ = 0.3; *p =* 0.563	0.005
Sociality		
Town	*F*_(1, 68)_ = 16.0; *p* < 0.0001	0.190
Time	*F*_(1, 68)_ = 1.1; *p =* 0.297	0.016
Time × Town	*F*_(1, 68)_ = 0.2; *p =* 0.631	0.003
Eating		
Town	*F*_(1, 68)_ = 1.1; *p =* 0.299	0.016
Time	*F*_(1, 68)_ = 0.2; *p =* 0.683	0.002
Time × Town	*F*_(1, 68)_ = 0.1; *p =* 0.749	0.002
Global Rhythms		
Town	*F*_(1, 68)_ = 5.9; *p* < 0.0001	0.081
Time	*F*_(1, 68)_ = 49.1; *p* < 0.0001	0.421
Time × Town	*F*_(1, 68)_ = 44.9; *p* < 0.0001	0.398

ANOVA analysis for repeated measures, shows no violation of the sphericity (Mauchly's *p* > 0.50). The repeated-measures ANOVA with a Greenhouse-Geisser correction determined that biological rhythms, when measured as the global score of the BRIAN scale, did not differ statistically significantly between time points [*F*_(1, 68)_ = 0.341, *p* = 0.561]. *Post-hoc* tests using the Bonferroni correction revealed that alterations in biological rhythms were higher in Cagliari than in Tunis (*P* < 0.0001; partial η^2^ = 0.30), without a town × time interaction [*F*_(1, 68)_ = 0.341, *p* = 0.979]. Essentially, alterations in biological rhythms were larger in Cagliari than in Tunis at both time points. In the MANOVA analysis for repeated measures, no violation of the sphericity was observed (Mauchly's *p* > 0.50 in all analyses). There was a statistically significant difference in the alteration of the biological rhythms based on town: [*F*_(5, 64)_ = 15.48, *p* < 0.0001]; Wilk's Λ = 0.453, partial η^2^ = 0.55.

There was also an effect of time and of time × town (Table 2).

Change over time depended essentially on an alteration in global rhythms. The results of the repeated measures MANOVA with a Greenhouse-Geisser correction showed that there was a change over time in global rhythms [*F*_(1, 68)_ = 49.34, *p* < 0.0001; partial η^2^ = 0.42] but not on the other dimensions (sleep, activity, social contacts, eating: *p* > 0.10 in all comparisons). The change over time in global rhythms also influenced the time per town interaction, inasmuch rhythms decreased over time in Tunis but increased in Cagliari [*F*_(1, 68)_ = 66.40, *p* < 0.0001; partial η^2^ = 0.40].

Overall, alterations in biological rhythms were higher in Cagliari than in Tunis at both time points. A change over time by town was observed in global rhythms but not in sleep, activity, social contacts, eating (Table 2).

Table 3 compares the biological rhythms within the Cagliari group during the lockdown in the subgroups with or without depression. The depressive sub-group, as expected, was more dysfunctional and was significantly worse statistically in the BRIAN scores in sleep, activity and nutrition. However, both groups showed a dominance of nocturnal biorhythm and a similar social rhythms. Table 4 compares the biological rhythms within the Cagliari group after the lockdown, the differences being confirmed even 2 months after the first evaluation.

**Table 3:** Biological Rhythms (Brian Scores) in the Cagliari sample according to the presence or absence of depressive symptoms during lockdown (t0).

	**Sleep**	**Activity**	**Social**	**Nutrition**	**Rhythms**	**Total**
Depression Positivity MEAN ± SD (*N =* 18)	14.38 ± 3.46	13.11 ± 3.51	7.84 ± 2.77	9.05 ± 2.83	5.72 ± 2.69	49.45 ± 14.9
Depression Negativity MEAN ± SD (*N =* 22)	10.81 ± 3.40	9.77 ± 4.79	7.63 ± 4.95	6.22 ± 2.35	5.42 ± 1.77	39.72 ± 7.44
*F*_(1, 38)_, df	10.618	5.904	0.025	11.917	0.941	7.215
*p*	0.002	0.020	0.876	0.001	0.338	0.01

**Table 4:** Biological Rhythms (Brian Scores) in the Cagliari sample distinguished according to the presence or absence of depressive symptoms after the lockdown (t1).

	**Sleep**	**Activity**	**Social**	**Nutrition**	**Rhythms**	**Total**
Depression Positivity MEDIA ± SD(*N =* 14)	14.14 ± 3.52	14.50 ± 3.85	7.57 ± 2.90	6.07 ± 2.46	6.21 ± 2.42	52.49 ± 14.6
Depression Negativity MEDIA ± SD (*N =* 26)	11.30 ± 3.07	10.42 ± 4.55	7.03 ± 2.73	5.67 ± 1.98	5.58 ± 2.00	41.07 ± 10.57
*F*_(1, 39)_, df	7.387	8.927	0.363	7.446	0.279	8.105
*p*	0.010	0.005	0.551	0.010	0.601	0.007

A one-way ANCOVA was conducted to compare the differences in biological rhythms by location (Cagliari and Tunis) whilst controlling for levels of depression. Levene's test and normality checks were carried out and the assumptions met [*F*_(1, 68)_ = 0.38, *p* = 0.54]. There was a significant difference in mean dysfunctional scores in the overall BRIAN scale [*F*_(1, 67)_ = 5.42, *p* = 0.023] at T1 between the towns. Comparing the estimated marginal means showed that the greatest alterations in biological rhythms were in Cagliari (44.1 ± 10.8) compared to Tunis (33.3 ± 8.3). This same effect was observed at T2 [*F*_(1, 67)_ = 7.04, *p* = 0.010], again with greater alterations in biological rhythms in Cagliari (44.9 ± 12.2) than compared to Tunis (34.1 ± 8.1).

## Discussion

The study suggests that patients with bipolar disorder being subjected to a rigid lockdown as in Cagliari and Italy in Spring 2020, showed higher frequency of depressive episodes and a modification in biological rhythms with impaired sleep, activity and social rhythms compared to people who, with the same disorders and at the same time, lived in Tunis during a less restrictive lockdown. The Tunis figure was within the expected range with respect to the results of a large cohort study recently published on bipolar disorders (31), while the incidence of depressive episode in Cagliari was significantly higher than the figure for the large cohort assumed as a normative (31).

Within the Sardinian sample, the characteristics of dysfunctionality in the biorhythms were associated with having an ongoing depressive symptomatology, i.e., the Cagliari patients with bipolar disorder and depression presented more severe impairment in the biorhythms of sleep, activity and nutrition than the Cagliari patients with bipolar disorder without depression in progress.

The higher frequency of depressive episodes in Cagliari may be a direct effect of the stress due to social isolation and poor contact during the quarantine, without a direct role of biorhythm dysregulation, eventually in sub-types of mood disorders with specific vulnerability to social isolation (32–34). In accordance with this point of view, the alterations of biorhythms may be the consequence of the depressive symptoms.

Alternatively, it may be hypothesized that the rigid lockdown caused a dysregulation of biorhythms and this would have favored depression. In this second hypothesis, depression is the consequence and not the cause of the alteration of the biorhythms associated with the rigid lockdown.

The fact that after the lifting of the lockdown the biorhythms remained unchanged in the Sardinian sample may be an argument in favor of the first hypothesis, but the time span that had elapsed is reasonably too short for a change to occur. While the evidence that people were selected in a situation of clinical stability and that depressive episodes arose (only in Cagliari and to a greater extent than in Tunis) during a stricter lockdown cannot exclude a selection bias in Cagliari of people with a specific vulnerability to depression from those of Tunis (in fact, it is to be noted that some characteristics of the two samples are not perfectly superimposable, i.e., gender frequency) and, above all, it cannot exclude the fact that the depression of mood preceded the dysregulation of the biorhythms. Through the ANCOVA analysis, however, the effects on the modification of the biological rhythm are independent of the co-presence of depressive symptoms. It can therefore be excluded that the alterations in biorhythms are a consequence of depressive symptoms. Thus, the data appear to suggest that the whole Sardinian sample was subjected to a common stressful trigger associated with an alteration of the biorhythms on the whole sample, causing only a few to have a depressive episode. Those who suffered from depression also experienced worse levels of altered biorhythms.

In terms of nutritional rhythms which instead present internal differences (Sardinians with depression vs. Sardinians without depression) but not external ones (Sardinians similar to Tunisians), it must be considered that most of the people of the Sardinian sample lived with family, where meals (in Sardinian but also generally in Mediterranean culture) remain occasions for sharing. It is therefore possible that the Sardinians (but also the Tunisians) who were not depressed were able to take advantage of this element of protection that may have been enhanced by the quarantine. This protective effect probably ceased when the threshold of the explosion of the depressive episode was exceeded and the depressive symptomatology caused a social distancing even in eating habits.

Evidence from the literature indicates that sleep disturbances may be a trigger of critical episodes in bipolar disorder (35, 36).

The pathophysiologically relevant elements in the mechanism of action of the triggering of episodes may be sleep-wake cycle interruptions and being subjected to artificial light pollution due to sleep dysregulation (37, 38). Although sleep dysregulation has been associated primarily with the onset of manic episodes in bipolar disorder, there is evidence that depression in bipolar disorder may also be caused by sleep dysregulation (35, 39). While in sub-threshold bipolar disorders sleep disturbances are one of the most frequent symptoms and often associated with subthreshold depression (40); sleep abnormalities are in fact indicated as a good predictor of a mood swing in Bipolar Disorder; the maintaining of a stable sleep-wake cycle was indicated as a key to the maintenance of stability (36).

This study found that rigid lockdown was associated with specific sleep dysregulations in bipolar patients even in people without current depressive episodes, but with greater impairment in people with depression. It may therefore suggest the validity of the second hypothesis mentioned above, i.e., that the lockdown introduced biorhythm dysregulation, and this made the subjects more vulnerable to depression.

In conclusion, the study suggests that a rigid lockdown may expose people with bipolar disorder to the risk of relapse through dysregulation of biological rhythms, and specifically sleep rhythms and to the risk of depressive disorder.

This element is confirmed as of great importance also from a public health point of view given the difficulties in support and access to care that has been reported by people with psychosocial disabilities during the lockdowns (41).

However, it must be specified that the exploratory nature of the study, given the complexity of the variables involved, does not allow a definitive verification of the starting hypotheses.

## Limits

This study has obvious limitations. Firstly, the time in which it was conducted did not allow for an extensive preparation which would have allowed researchers to define greater predictive homogeneity criteria as well as the collection of larger samples. Although the assessment of stability prior to lockdown was made by clinical assessment, it was not standardized.

This study compared two cohorts of patients having cultural differences (42) which may also have influenced the possible confounding factors, such as the fear of the pandemic as a trigger of episodes, the greater impact related to certain deprivations induced by quarantine (minor social supports, less access to religious ceremonies, etc.).

The study pre-selected two samples that gave stable, symptom-free results in clinical evaluation prior to lockdown. This poses two series of problems: firstly, the imperfect reliability of the routine clinical evaluation carried out by the two teams with different psychiatric training and culture (albeit for years in collaboration); secondly, it selected a particular type not representative of all patients with bipolar disorder, many of whom have chronic symptoms.

Another limitation is that a return to a more functional biorhythm after the lockdown has not been shown over time. Although this is reasonable even if we accept the hypothesis that it was the lockdown that caused the alteration of the biorhythms, given the limited time that has elapsed, unfortunately the rekindling of the pandemic and the restoration of new restrictive measures both in Cagliari and Tunis will prevent, at least in the short term, verification of the beneficial effect of the return to normality on the two cohorts. Future studies with larger samples and longer observational times including the return to normality are needed to verify the hypothesis.

Finally, our study could not investigate the possible differences in the incidence of manic episodes in the two samples owing to its low power consequent to the small sample and the limited time of observation, This aspect will therefore require future research, also in the light of the results of our study on depressive episodes.

## Conclusions

A rigid lockdown may expose people with BD to depressive relapse through dysregulation of biological rhythms. The return to more functional rhythms did not appear 1 month after lockdown. The rekindling of the pandemic and the restoration of new restrictive measures will prevent, at least in the short term, the beneficial effect of a return to normality in the two cohorts.

This was a limited exploratory study; future studies with larger samples and longer observational time to verify the hypothesis are needed.

## Data Availability Statement

The raw data supporting the conclusions of this article will be made available by the authors, without undue reservation.

## Ethics Statement

The studies involving human participants were reviewed and approved by Comitato Indipendente della Azienda Ospedaliero Universitaria di Cagliari and by the Ethics Committee of Razi Hospital La Manouba. The patients/participants provided their written informed consent to participate in this study.

## Author Contributions

MC and UO had the first idea of the survey (MC and UO were also responsible for the database) that was planned jointly with APe, AB, LB, AAi, AAr, SL, GC, and FN. The study and data processing were conducted with the participation of all the authors. AB coordinated the data collection in Tunis and APe in Cagliari. MC, UO, and FN wrote the first draft that was revised by each of the participants who approved the reworked paper based on the suggestions. The work was then re-discussed with APr. APr and MC conducted further analysis and modified the first draft. The final draft was re-discussed and shared by all authors. All authors contributed to the article and approved the submitted version.

## Conflict of Interest

The authors declare that the research was conducted in the absence of any commercial or financial relationships that could be construed as a potential conflict of interest.
